# Neural predictors of gait stability when walking freely in the real-world

**DOI:** 10.1186/s12984-018-0357-z

**Published:** 2018-02-27

**Authors:** Sara Pizzamiglio, Hassan Abdalla, Usman Naeem, Duncan L. Turner

**Affiliations:** 1 Neuroplasticity and Neurorehabilitation Doctoral Training Programme, Neurorehabilitation Unit, School of Health, Sport and Bioscience, College of Applied Health, University of East London, E15 4LZ, London, UK; 2 School of Architecture, Computing and Engineering, University of East London, University Way, London, UK; 3 UCLP Centre for Neurorehabilitation, London, UK

**Keywords:** Mobile brain/body imaging (MOBI), EEG, Gait, Acceleration, RMSR, Urban environment

## Abstract

**Background:**

Gait impairments during real-world locomotion are common in neurological diseases. However, very little is currently known about the neural correlates of walking in the real world and on which regions of the brain are involved in regulating gait stability and performance. As a first step to understanding how neural control of gait may be impaired in neurological conditions such as Parkinson’s disease, we investigated how regional brain activation might predict walking performance in the urban environment and whilst engaging with secondary tasks in healthy subjects.

**Methods:**

We recorded gait characteristics including trunk acceleration and brain activation in 14 healthy young subjects whilst they walked around the university campus freely (single task), while conversing with the experimenter and while texting with their smartphone. Neural spectral power density (PSD) was evaluated in three brain regions of interest, namely the pre-frontal cortex (PFC) and bilateral posterior parietal cortex (right/left PPC). We hypothesized that specific regional neural activation would predict trunk acceleration data obtained during the different walking conditions.

**Results:**

Vertical trunk acceleration was predicted by gait velocity and left PPC theta (4–7 Hz) band PSD in single-task walking (R-squared = 0.725, *p* = 0.001) and by gait velocity and left PPC alpha (8–12 Hz) band PSD in walking while conversing (R-squared = 0.727, *p* = 0.001). Medio-lateral trunk acceleration was predicted by left PPC beta (15–25 Hz) band PSD when walking while texting (R-squared = 0.434, *p* = 0.010).

**Conclusions:**

We suggest that the left PPC may be involved in the processes of sensorimotor integration and gait control during walking in real-world conditions. Frequency-specific coding was operative in different dual tasks and may be developed as biomarkers of gait deficits in neurological conditions during performance of these types of, now commonly undertaken, dual tasks.

## Background

Recent developments in mobile technologies enable the design of experiments describing behavioural and neural responses of subjects performing commonly observed tasks in real-world scenarios outside of the experimental lab environment [1]. Such tasks may include artistic performance such as dancing and music playing [2], dealing with stressful situations [3] and evaluating changes in the levels of “excitement”, “engagement” and “frustration” when walking within different city areas [4, 5]. An interesting aspect of these novel experimental approaches is the possibility to correlate brain activity and natural behaviour, in both healthy and neurologically impaired populations [1]. For example, recent evidence has suggested that the pre-frontal cortex (PFC) is involved in multitasking behaviours [6–8] and that the posterior parietal cortex (PPC) is engaged in motor adaptation during walking in health [9–11]. These regions have also been shown to be involved in different attentional [12] and executive function networks [13]. Gait initiation failure (GIF) and freezing of gait (FoG) episodes in freely walking Parkinson’s disease (PD) patients have been correlated with increased neural activity and connectivity between different cortical regions such as occipital, parietal and frontal regions [14, 15]. Clinically, difficulties in free walking are observed to increase with the severity of PD due to damage in the cortical-striatal locomotor network [16]. Ambulatory abilities of PD patients are impaired by muscular hypertonia and hypokinesia, which induce asymmetries and reduce speed, as well as FoG [17]. PD patients have less control of their posture when standing, walking and compensating for an external perturbation and this may lead to an increased magnitude of postural sway [18]. Specifically, the magnitude of medio-lateral sway was shown to be highly sensitive to postural impairments during both standing and over-ground free walking and this progressed with the severity of PD [19, 20].

In ths study, we used a smartphone to measure the acceleration root mean square index (RMS) as an indication of the magnitude of movements or sway at the pelvis in any of the three movement directions (i.e., vertical, antero-posterior and medio-lateral) [18, 21–23]. Previous investigations have shown that RMS increases at the level of the pelvis when walking on an insidious surface (i.e., more difficult) compared to smooth conditions, but not at the head [21, 24]. Normalization procedures have also been developed for RMS data to reliably compare the quality and variability of real-world gait between different populations (healthy young vs. elderly vs. neurologically impaired) and at different gait speeds [22, 25–27].

Whilst RMS has been correlated with age or level/type of neurological impairments, there have been no models of how neural activation can predict gait stability [20]. We hypothesised that in healthy young subjects, neural activity in the PFC and PPC regions would predict gait stability, specifically measured with the acceleration RMS index. To test our hypothesis, we investigated the relationships between neural activity and RMS index during different ambulatory conditions outside the laboratory using real life tasks. We studied three common ambulatory tasks, namely self-paced free walking, walking whilst conversing and walking whilst texting on a smartphone in order to better understand the neural correlates underlying human natural behaviours.

## Methods

### Ethical approval

Eighteen right-handed healthy young adults (age mean ± standard deviation (SD) = 25 ± 3, 7 male/11 female] with no previous history of neurological, musculoskeletal or gait disorders, volunteered for the study by giving written informed consent. The study was approved by the University of East London Ethics Committee (UREC_1415_29) and all experiments were conducted in accordance with the Declaration of Helsinki. Data of three subjects were discarded because of technical failures during data acquisition (two males, one female) and of one subject (female) because of poor data quality, leaving a total of 14 subjects (age mean ± SD = 26 ± 3, 5 male/9 female].

### Experimental protocol

Subjects were first prepared in a laboratory room and then guided outside into the campus garden [28]. During this period, no signals were recorded and subjects were instructed to become familiar with the setup and communicate to the experimenter if anything was not properly set. Once outside, subjects were shown the predefined walking path (200 m) and were instructed to walk at their preferred natural speed, as previous studies have shown that gait behaviour and patterns are optimized when walking at the natural speed [21, 26]. Experiments consisted of three conditions during which subjects walked along the predefined path naturally (Single-Task, ST), while conversing with the experimenter (Dual-Task1, DT1) or texting with their smartphone (Dual-Task2, DT2). The dual-task conditions were randomized across subjects in order to avoid bias in gait behaviour and recordings. The dual-task conditions were designed to represent real-life situations and, to standardise them, conversations during DT1 were based on a set of standard questions, whereas in DT2 subjects read and replied to a standard email. Subjects rested for a period of 5 min after each condition in order to avoid fatigue. The experimenter followed the subjects during each condition recording videos of gait behaviour. Experiments were carried out only during dry days free from strong winds and/or rain.

### Recording techniques

**Fig. 1:**
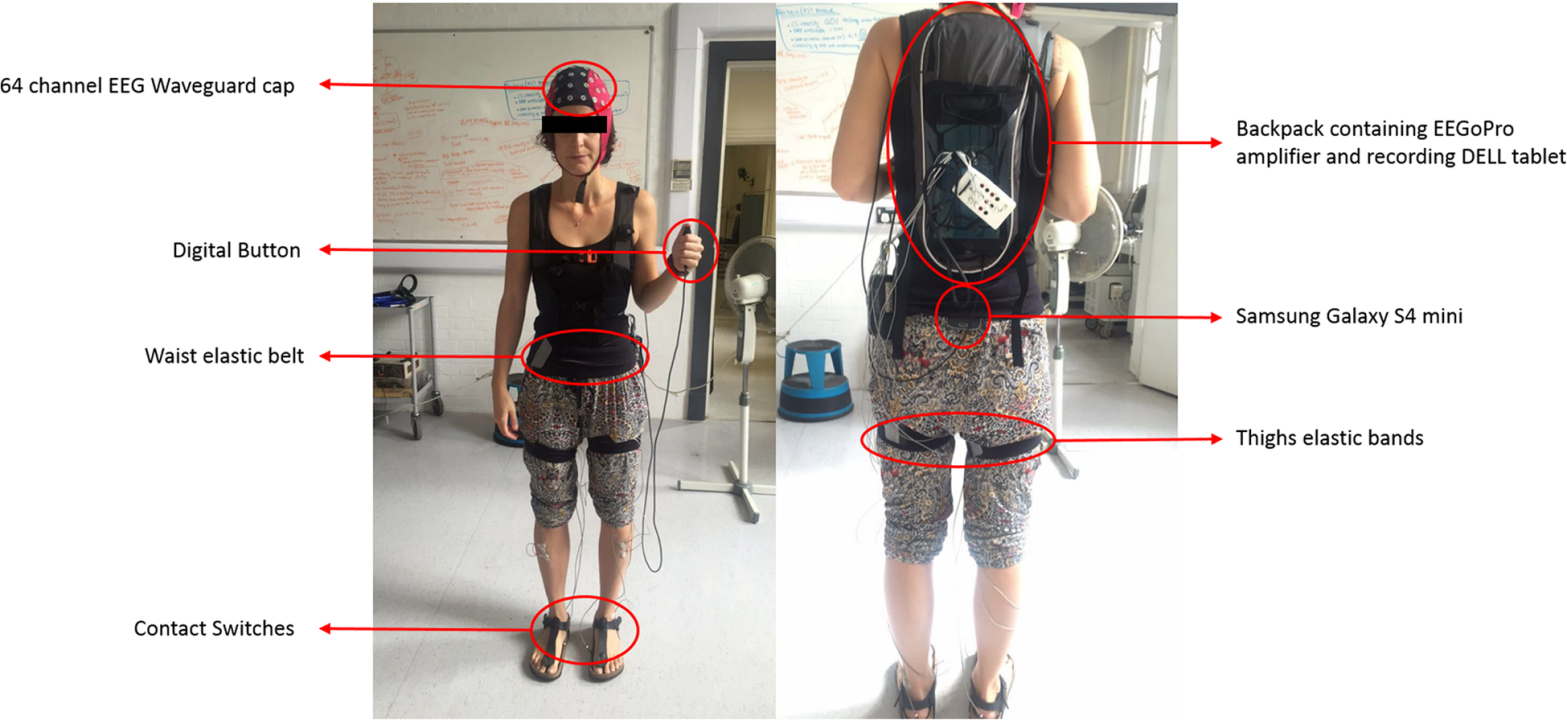
Mobile Setup for real-world experiments. Brain activity was recorded by a 64 channel EEG Waveguard cap connected to the EEGoPro amplifier placed into a backpack together with a tablet on which the recording software ran. Contact Switches were placed underneath the subject’s heels and connected to a digital input of the MWX8 DataLog analog-to-digital converter fixed at the subject’s hips by an elastic belt. Elastic bands were also placed around the subject’s thighs to make sure cables did not disturb gait performance. A digital button was connected to the converter and pressed by the subject at specific time points. A Samsung Galaxy S4 mini was firmly placed at the subject’s lower back with the elastic belt. Author S.P. gave written informed consent for the usage of this picture

The experimental setup, illustrated in Fig. 1, is fully mobile and allows the recording of physiological and behavioural data during walking (or any other mobile situation). Brain activity (EEG; μV) was continuously recorded via a high-density 64 channel Waveguard cap (ANT Neuro, Enschede, Netherlands) with an EEGoPro amplifier (ANT Neuro, Enschede, Netherlands) at a sampling frequency of 1 kHz and raw signals filtered between 0.1 and 500 Hz. Impedances were kept below 5 kΩ for the whole duration of the experiment and data were referenced to the FCz channel. The recording EEG amplifier was carried by the subject within a backpack. A Samsung Galaxy S4 mini smartphone was fixed at the subject’s lower back with an elastic belt and data from its internal accelerometers and gyroscope were recorded through the AndroSensor app at a frequency of 200 Hz. The lower back position is the currently most preferred and reliable location to observe changes in gait patterns across different conditions and populations [22]. Two digital force sensing resistor sensors (FSRs) were employed as contact switches and fixed underneath the subject’s heels to detect times of heel strikes. Data were recorded at 1 kHz by a 14 bit analog-to-digital converter (DataLog MWX8, Biometrics Ltd., Newport, UK) fixed at the subject’s hip by an elastic belt. These sensors return a digital binary output where the active edge is set 1-to-0, i.e., 0 when the heels make contact with the ground. A digital button (1-to-0 active edge) was also connected to the converter and pressed by the subject for 5 seconds at the beginning and at the end of each condition to define time points of start and finish. Elastic bands were placed around the subject thighs to fix the cables of contact switches to avoid uncomfortable situations and prevent the subject from falling/stepping on them. To synchronize data from the digital sensors representing important time points (i.e., start, heel strikes, end) with physiological variables, a common train of 12 consecutive TTL pulse was simultaneously sent to both the DataLog MWX8 converter and the EEGoPro amplifier at the beginning and at the end of the experiment. The matching of start and end points of each single pulse between the recordings were checked offline and eventually used as milestones for realigning the signals’ time axes.

### Data analysis

#### Gait measures

Gait measures were analysed with the iGAIT toolbox for MatLab [29] which provides a simple user-friendly GUI to display and analyse gait linear acceleration data recorded by different types of accelerometers. Features extracted by trunk accelerometery have been previously shown to successfully provide valuable insights on both healthy and pathological gait patterns and to be a potential discriminator of gait quality among populations [30]. Spatio-temporal and frequency features of gait were extracted and further used such as stride duration (sec), mean step length (m), velocity (m/s), step regularity in the vertical and antero-posterior directions, acceleration root mean square (RMS) in each movement direction (i.e., vertical (ver-), medio-lateral (ml-) and antero-posterior (ap-). To estimate gait stability across our experimental conditions, we employed a measure of normalized RMS (the RMS Ratio; RMSR) according to the formula:

$$ { RMS R}_x=\frac{RMS_x}{\sqrt{RMS_{AP}^2+{RMS}_{Ver}^2+{RMS}_{ML}^2}} $$

where x = ver-, ap- and ml- directions of acceleration. RMSR_x_ represents the ratio between the RSM in each direction and the overall RMS magnitude. These measures are representative of the quality and/or abnormality of gait [26] and used here to assess correlations with neural activation. However, it has also been demonstrated that velocity influences gait behaviour in both healthy and neurological populations [23, 26, 31]. Consequently, velocity was included as a factor in the model analysis of the correlations between neural activity and gait behaviour.

#### EEG pre-processing

EEG data were pre-processed using EEGLab open source toolbox for MatLab [32]. Data were first band-pass filtered between 0.5 Hz and 100 Hz (FIR filter, order automatically set by EEGLab) to minimize slow drifts and remove high-frequency components and then notch filtered at 50 Hz (FIR notch filter, order = 3302) to remove the power line noise. Visual inspection was performed on continuous data in order to identify and temporarily discard EEG channels affected by major noise sources throughout the whole experiment and to permanently remove data affected by prominent artefacts across all the recording channels. Data were then re-referenced to the common average reference and decomposed using independent component analysis (ICA) with the extended Infomax algorithm as implemented in EEGLab [33, 34]. Power spectral, spatial and temporal features of each independent component (IC) were carefully inspected and those representing typical artefacts (e.g. eye blinks, saccades, neck muscles) were removed from the data and remaining components were projected back to the scalp. Previously removed bad channels were then interpolated [35, 36] and all data then re-referenced again to the common average reference. Continuous data were then segmented into epochs of 1.8 s duration from − 200 ms to 1600 ms around each right heel strike in order to capture a complete stride (composed by, in order: right, left, right heel strikes) even at the slowest speed. One last visual inspection was performed to check the quality of the cleaned data and eventually remove still noisy epochs.

#### Electrodes regions of interest (ROI)-based power spectral density (PSD)

**Fig. 2:**
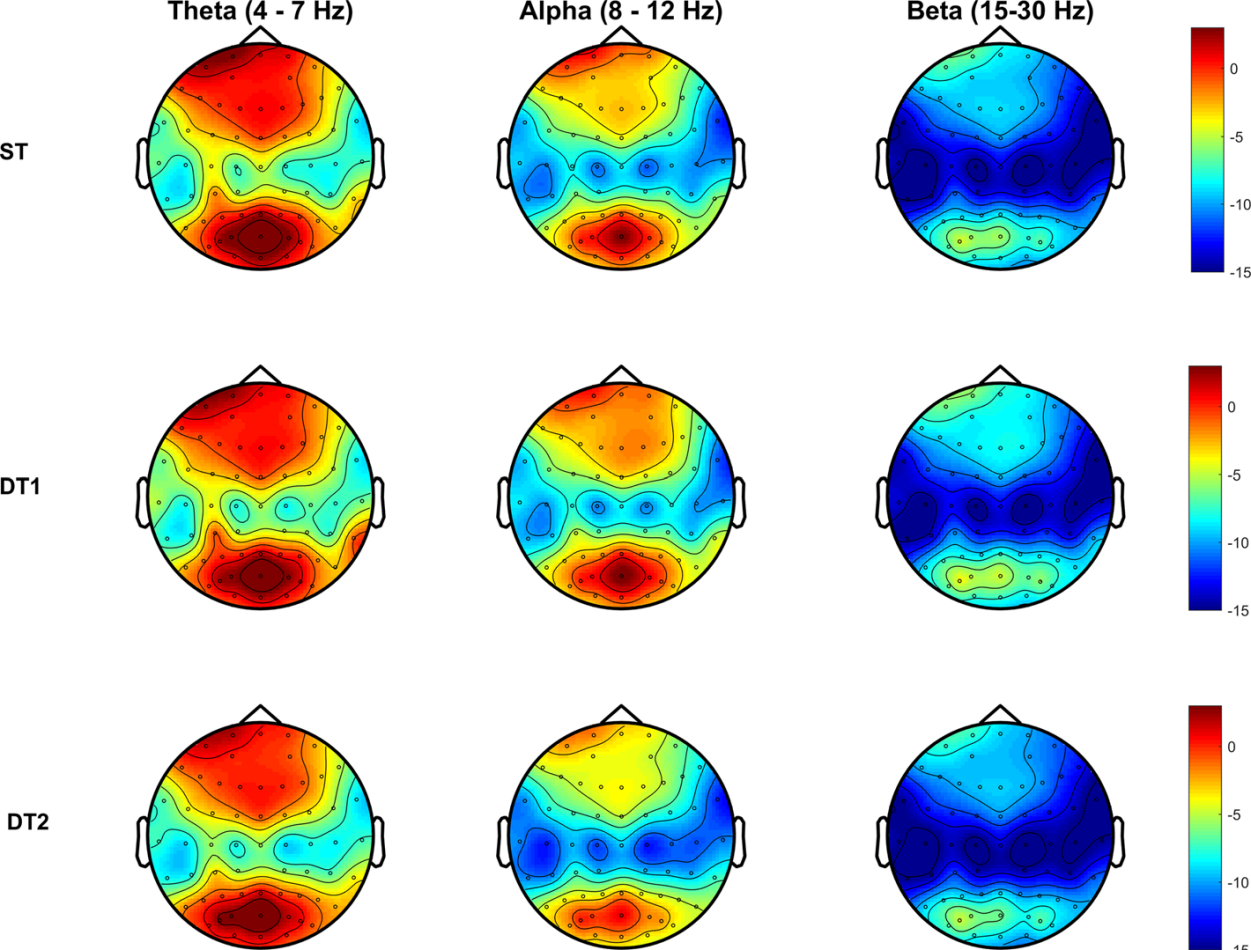
Power Spectral Density (PSD) across conditions. The spectral power of one typical subject in each condition (ST = Single-Task walking; DT1 = Dual-Task walking while conversing; DT2 = Dual-Task walking while texting with a smartphone) and for each frequency band of interest (θ, α and β). Values are colour-coded and expressed in dB. Subject P.S. gave written informed consent for the publication of her data

PSD was calculated through the EEGLab function *spectopo* in the frequency domain of each channel, in each frequency band of interest for each subject. This function uses the Welch’s overlapped segment averaging estimator as implemented in MatLab (*pwelch()* function) to calculate the PSD. A default Hamming window of 400 ms with a 50% overlap (i.e., 200 ms) was adopted and PSD for frequencies from 2 Hz to 50 Hz calculated. Figure 2 shows the whole-brain PSD activity of a typical subject across conditions and for each frequency band of interest (FOI, θ (4–7 Hz), α (8–12 Hz), β (15–30 Hz)). Further statistical analyses focused only on three ROIs, as we hypothesised a change in gait behaviour would be related to changes in brain activities in those areas mostly employed during executive functions, spatial attention and navigation, sensorimotor integration and adaptation during multi-tasking. Previous findings have localized consistent brain activities during both single-task and dual-tasks as well as adaptation in walking in PFC and PPC clusters [10, 11, 37]. We therefore defined three ROIs as: PFC including electrodes FP1, FPZ, FP2, AF7, AF3, AF4, AF8 and specifically laying over the Brodmann areas 9, 10 and 46, namely the dorsolateral and anterior prefrontal cortex thought to be involved in high-order executive functions (https://www.trans-cranial.com/); the left PPC including electrodes P7, P5, P3, PO3, PO5, PO7 and the right PPC including electrodes P8, P6, P4, PO4, PO6, PO8, which together cover bilaterally Brodmann Areas 7, 19, 37 and 39, namely the associative visual cortex, the parietal-occipital-temporal lobe and the angular gyrus, thought to be involved in high-cognitive functions (sensorimotor integration) and cross-modal association amongst somatosensory, auditory and visual information inputs (https://www.trans-cranial.com/). For each ROI, the average PSD value among the ROI-specific electrodes was calculated in each FOI, for each condition separately and for each subject.

### Statistics

Statistical analyses were all run with SPSS 23 software (IBM).

#### Gait measures

Gait measures were first calculated separately for each subject in each condition and group-level differences between conditions were assessed statistically. The Kolmogorov-Smirnoff test for normality was used to test the distribution of the data. Data were all normally distributed, thus parametric statistical tests were further employed. One way repeated measures ANOVA with factor “Condition” (three conditions, α = 0.05) was applied to each gait measure of interest to identify variance differences across conditions. Subsequently, paired samples T-tests with Bonferroni correction for multiple comparisons were employed to specifically define differences between conditions. T-tests significance level was set at α = 0.05. The total number of tested comparisons (i.e., repeated measures) was three, which specifically compared ST vs. DT1, ST vs. DT2 and DT1 vs. DT2. According to Bonferroni correction for multiple comparisons, the adjusted α level below which a comparison could be considered as statistically significant was 0.05/3 = 0.0167.

#### Predictive models of gait behaviour using PSD

Predictive models were created for each experimental condition (i.e., ST, DT1 and DT2) separately considering acceleration RMSR in each direction (i.e., vertical, antero-posterior and medio-lateral) as diverse Dependent Variables (DVs). Thus, for each acceleration RMSR direction, three condition-specific models were generated. In each condition-specific model, the related PSD in each FOI (× 3) and ROI (× 3) were considered as independent variables (IVs, i.e., predictors) together with gait velocity for a total of 10 IVs. Multiple Regression models were created with the format:

$$ {RMSR}_x={\beta}_0+{\beta}_1\cdot Velocity+{\beta}_{ij}\cdot PSD\_{ROI}_{i\_}{FOI}_j+\varepsilon $$

whereby RMSR_x_ is the DV and represents the acceleration RMSR in the direction x (i.e., vertical, antero-posterior, medio-lateral), and the IVs are Velocity and PSD_ROI_i__FOI_j_, which represents the PSD in the ROIi (i.e., frontal, right-parietal, left-parietal) and in the FOIj (θ, α, β). IVs were entered stepwise into the model, which means that only the IVs significantly correlating with the DV were added into the final model. *β*_*n*_ are the intercept and coefficient associated to each model predictor, and ε is the error. Data were first centred (i.e., the mean score was subtracted from each observation) and scaled (i.e., SD was set equal to 1) in order to reduce the chance of multicollinearity. Measures of Goodness-of-Fit (B) and *p*-values associated to each significant predictor are presented.

## Results

### Gait measures across conditions

**Fig. 3:**
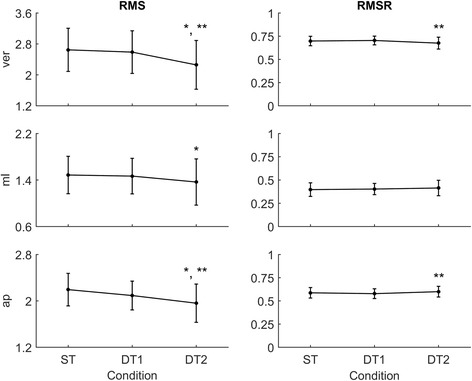
Acceleration RMS and RMSR profiles across conditions. Condition-by-condition population average (*N* = 14) profiles with standard deviation error bars for each movement direction (ver = Vertical, ml = Medio-Lateral, ap = Antero-Posterior). Statistically significant paired-samples t-test corrected for multiple comparisons (Bonferroni, × 3) are highlighted with * (ST vs DT_i_ with i = 1, 2) and/or ** (DT1 vs. DT2). Detailed results are reported in Table 1. Average acceleration RMS decrease in the two dual-task conditions with respect to the single-task condition regardless of movement direction. Average acceleration RMSR decrease in the vertical direction and increase in the medio-lateral and antero-posterior directions decrease in the two dual-task conditions with respect to the single-task condition regardless of movement direction

Subjects walked along the predefined path of 200 m in an average time of 225 ± 25 s during single-task (ST) walking and significantly more slowly when walking while conversing (DT1; 235 ± 28 s) and when walking while texting (DT2; 260 ± 41 s; RMANOVA F = 21.660, *p* < 0.001). Performing any secondary tasks significantly increased the walking time with respect to ST (ST vs. DT_i_ with i = 1,2 all t < − 3.906, all *p* < 0.002) and walking while texting required a longer time compared to walking while conversing (DT2 vs. DT1, t = − 3.993, *p* = 0.002). Velocity was significantly slower (RMANOVA F = 34.215, *p* = 0.001) in both DT1 (t = 4.199, *p* = 0.001) and DT2 (t = 6.847, p = 0.001) compared to ST and slower in DT2 vs DT1 (t = 4.991, *p* = 0.001; see Table 1). Gait measures of stride duration, ver-RMS and ap-RMS were statistically different across conditions (RMANOVA F > 12.165, *p* < 0.001), specifically between DT2 vs. ST (t > (−) 3.503, *p* < 0.004) and DT2 vs. DT1 (t > (−) 3.793, *p* < 0.002). Gait measures of ml-RMS and ap-step regularity significantly differed across conditions (RMANOVA F > 4.559, *p* < 0.043), specifically between DT2 vs. ST (t > 2.772, *p* < 0.016). Gait measures of ver-RMSR and ap-RMSR also changed significantly across conditions (RMANOVA F > 3.990, *p* < 0.031), specifically between DT2 vs. DT1 (t > 3.618, *p* < 0.003). No significant differences were found for measures of mean step length, ml-RMSR and ver-step regularity (RMANOVA F < 3.099, *p* > 0.05). Measures of acceleration RMS/RMSR in each direction across the three conditions are represented in Fig. 3.

**Table 1:** Single- and dual-task gait measures. Condition-by-condition mean (± SD) measures of gait performance (*N* = 14)

	Single Task	Dual Task 1	Dual Task 2	Anova F	Anova p
Stride Duration (ms)	1054 (± 87)	1060 (± 79)	1106 (± 107)*, **	12.165	0.001
Mean Step Length (m)	0.53 (± 0.06)	0.52 (± 0.08)	0.51 (± 0.07)	0.0769	N.S.
Velocity (m/s)	0.90 (± 0.10)	0.86 (± 0.10)*	0.78 (± 0.12)*, **	34.215	0.001
ver-RMS	2.65 (± 0.56)	2.59 (± 0.55)	2.26 (± 0.63)*, **	17.554	0.001
ml-RMS	1.48 (± 0.32)	1.47 (± 0.31)	1.37 (± 0.40)*	7.769	0.008
ap-RMS	2.19 (± 0.28)	2.09 (± 0.25)	0.96 (± 0.33)*, **	16.946	0.001
ver-RMSR	0.70 (± 0.05)	0.70 (± 0.05)	0.67 (± 0.06)**	5.839	0.008
ml-RMSR	0.40 (± 0.07)	0.40 (± 0.06)	0.41 (± 0.08)	1.735	N.S.
ap-RMSR	0.59 (± 0.06)	0.58 (± 0.053)	0.60 (± 0.06)**	7.165	0.003
ver-Step Regularity	0.75 (± 0.09)	0.69 (± 0.19)	0.69 (± 0.13)	2.027	N.S.
ap-Step Regularity	0.76 (± 0.09)	0.73 (± 0.11)	0.71 (± 0.09)*	6.642	0.005

Repeated measures ANOVA *p*-values are reported in the right-side column. Statistically significant paired-samples t-test corrected for multiple comparisons are highlighted with * (ST vs DT_i_ with i = 1, 2) and/or ** (DT1 vs. DT2). N.S., not significant

### Predicting single-task walking stability using neurophysiological activity

**Fig. 4:**
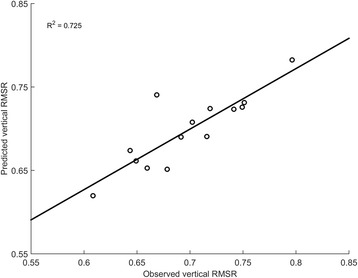
Observed vs. Predicted ver-RMSR values according to the multiple regression model during ST. The model R-squared value associated to the line of fit of the model in the figure

A regression model was successfully created for ver-RMSR (R-squared = 0.725, *p* = 0.001) for which all assumptions were met (no multicollinearity, no auto-correlation, no homoscedasticity). Only the IVs significantly contributing to the prediction of ver-RMSR were entered stepwise into the model, which predicted the DV based on Velocity (B = 0.355, *p* = 0.001) and left-parietal theta PSD (B = 0.009, *p* = 0.026) as shown in Fig. 4. The final model equation is reported in Table 2. Multiple linear regression analysis did not provide statistically significant models for ml-RMSR or ap-RMSR during single-task walking.

**Table 2:** Prediction models. Condition-by-condition prediction models for each acceleration RMSR direction. NA defines cases in which no statistically significant and/or reliable model (i.e., for which all assumptions were met) was created

	Dependent Variables		
Conditions	RMSR_ver_	RMSR_ap_	RMSR_ml_
ST	0.699 + 0.355 ⋅ *Velocity* + 0.009 ⋅ *left Parietal θ PSD*	NA	NA
DT1	0.704 + 0.029 ⋅ *Velocity* + 0.021 ⋅ *left Parietal α PSD*	NA	NA
DT2	NA	NA	0.414 − 0.055 ⋅ *left Parietal β PSD*

### Predicting dual-task walking stability using neurophysiological activity: walking while conversing

**Fig. 5:**
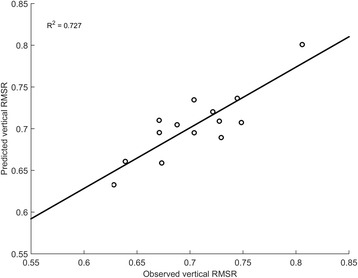
Observed vs. Predicted ver-RMSR values according to the multiple regression model during DT1. The model R-squared value associated to the line of fit of the model in the figure

A regression model was successfully created for ver-RMSR (R-squared = 0.727, *p* = 0.001) for which all assumptions were met (no multicollinearity, no auto-correlation, no homoscedasticity). Only the IVs significantly contributing to the prediction of ver-RMSR were stepwise entered into the model, which predicts the DV based on Velocity (B = 0.029, *p* = 0.003) and left-parietal alpha PSD (B = 0.021, *p* = 0.020) as shown in Fig. 5. The final model equation is reported in Table 2. Multiple linear regression analysis did not provide statistically significant models for ml-RMSR and ap-RMSR when walking while conversing.

### Predicting dual-task walking stability using neurophysiological activity: walking while texting with a smartphone

**Fig. 6:**
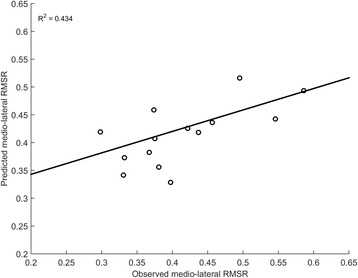
Observed vs. Predicted ml-RMSR values according to the multiple regression model during DT2. The model R-squared value associated to the line of fit of the model in the figure

A regression model was successfully created for ml-RMSR (R-squared = 0.434, *p* = 0.010) for which all assumptions were met (no multicollinearity, no auto-correlation, no homoscedasticity). Only the IVs significantly contributing to the prediction of ml-RMSR were stepwise entered into the model, which predicts the DV based only on left-parietal beta PSD (B = − 0.055, *p* = 0.010), as shown in Fig. 6. The final model equation is reported in Table 2. Multiple linear regression analysis did not provide statistically significant models for ver-RMSR and ap-RMSR when walking while texting.

## Discussion

### Novel findings

We observed that region- and frequency- specific brain activation could predict gait stability during free self-paced walking and walking during dual-tasking. Two walking conditions (i.e., single-task walking and walking while conversing) were characterised by a positive relationship between ver-RMSR, velocity and left parietal PSD in θ and α frequency bands respectively. On the other hand, walking while texting featured a negative relationship between ml-RMSR and left parietal PSD in the β frequency band. Thus the left PPC appears to play a general role in walking in the real-world and the type of secondary task may be correlated to frequency-specific coding in the left PPC.

### Relationship between brain and behaviour

We tested whether prefrontal and bilateral posterior parietal spectral activity could relate to gait behaviour as it has been shown that these are the most modulated cortical areas during dual-task free walking [6, 11, 37, 38] and in dual-task laboratory-based experiments [39]. No linear relationship was found between the PFC and gait stability, despite the great interest in this brain region when monitoring multitasking ambulatory activities [6, 7, 37, 40–43]. On the contrary, the left PPC was shown to be related to gait behaviour regardless of the secondary task type undertaken. The left PPC has been extensively studied in laboratory-based experiments both on animals and humans [44]. Currently, left PPC is thought to act as the sensorimotor integrator and rapid online updater of movement planning [45], integrating spatial information of the surroundings and sensory feedback with motor planning and executive commands [44]. First evidence of the involvement of the PPC in inter-limb coordination during walking was obtained through intra-cortical recordings of visually-guided walking cats [46]. It was postulated that PPC neurons were involved in the regulation of inter-limb coordination during locomotion requiring visual guidance, thus playing a role in processes of sensorimotor integration (i.e., visuomotor integration) [47]. In humans, PPC is believed to be involved in the integration of sensory information with motor plans, which is severely impaired in PD patients with FoG [48]. Indeed, the spatial navigation deficits seen after damage of bilateral PPC as in Balint’s syndrome [49] or neglect [50] arise because of the inability to integrate spatial orientation with current/future planning of the voluntary movement needed to accomplish the end goal. If the PPC is in general believed to play such a complicated role, then each hemispheric area has been characterized with specific roles. The right-PPC, in connection with frontal regions, is strongly involved in multiple types of attention [12, 51]. On the other hand, the left PPC has been recently shown to play a major role as a sensorimotor integrator as PD patients that exhibit FoG display reduced functional connectivity between left PPC and multiple brain regions such as the somatosensory and auditory areas [52]. Left PPC is therefore suggested to work as a sensorimotor integrator during online movement planning and monitoring, whereas the right PPC actively engages with different attentional networks. In summary, we suggest that left PPC plays a primary active role in monitoring and planning the walking movement regardless of any secondary conditions undertaken in parallel. However, the different relationships between neural activity in this brain region and changes in gait stability across walking conditions are encoded by different frequency-specific activity.

### Task-specific relationships between brain activation and gait behaviour

When walking naturally, a positive linear predictive relationship could be identified between left PPC activity in the θ frequency band and ver-RMSR and velocity. Ver-RMS(R) has been shown to depend linearly on gait speed and to represent the quality of gait, with higher values symbolic of a more stable and rapid walk (typical of healthy young adults) and lower values symbolic of reduced flexibility and bent postures (typical of older adults) [22, 23, 26]. We confirmed the positive relationship between ver-RMSR and velocity, but added a predictive neural component (left PPC). When walking naturally without engaging in any secondary tasks, left PPC has been shown to be active in the θ range (4–7 Hz) [53–55] and is believed to be connected to deeper structures such as the hippocampus actively engaged in the navigation process [56].

When walking while conversing, a positive predictive relationship could be identified between left PPC activity in the α frequency band and ver-RMSR and again velocity. As θ neural oscillations likely engage in memory retrieval and organization of ‘thoughts’ when subjects are engaged in a conversation [57, 58], is it likely that higher frequency oscillations (i.e., α) took over the duty of sensorimotor integrator and monitor during walking in the present study. This hypothesis is further supported by recent studies showing involvement of α (8–12 Hz) oscillations in spatial navigation [55, 59] and sensorimotor integration during walking speed adaptation to an external pace cue [11].

A significant negative predictive relationship was found between left PPC spectral activity in the β frequency band (15–30 Hz) and ml-RMSR when walking while texting. ml-RMSR has been recently validated as a marker of gait abnormality and recovery, where abnormally high values of trunk acceleration decrease alongside recovery [26, 27]. Moreover, stronger β desynchronization is required in arduous conditions in order to maintain the ‘status quo’ and to promote the voluntary action in a more challenging dual-task context [9–11, 60]. The negative relationship suggests that those subjects showing higher gait variability in the medio-lateral direction required stronger β desynchronization in order to accomplish the simultaneous tasks. This is in line with previous studies that showed that β PSD in the posterior parietal and occipital areas could be used to reliably classify and detect events such as FoG and GIF in Parkinson’s disease patients [14, 15], thus confirming the involvement of this brain region in postural and movement online control via sensorimotor integration. Of note, ml-RMSR did not correlate with velocity in this study as previously reported in the literature [26].

### Limitations and future perspective

In summary, spectral activity in the left PPC can predict stability of gait during different walking conditions through frequency-specific neural mechanisms. As the healthy population sample was young and small in number, the study could be expanded to include healthy older adults and PD patients. The inclusion of healthy older adults and PD patients would enable further testing of the hypothesis that the left PPC is crucially involved in the underlying mechanisms of successful walking in the presence of complex secondary tasks. This would be important in order to observe how specific neural activities can predict altered gait patterns due to cognitive decay and/or neurological impairments.

The analyses performed in this study were limited to the sensor-level (i.e., electrode data) and in the absence of subject-specific anatomical MRI data and care was taken not to over-stretch data interpretations towards the sources of neural activity. Future studies would benefit from anatomical MRI data for each subject or patient in order to reliably source the origins of neural activity especially as brain anatomy varies with age and stage of PD [61]. Lastly, both dual-task conditions required the subjects to share part of their attention to the ambulatory task with a second task, and difficulties in reallocating attentional resources could have contributed to the decrease in gait stability [39]. Future studies should therefore identify changes in level of attention as potential covariates in the predictive relationship between brain activations and gait behaviour using recent developments in MOBI of locomotion in health and disease [62, 63].

## Conclusions

In this study, we demonstrated that region- and frequency-specific brain activity could predict gait stability in several commonly undertaken tasks whilst walking in an urban environment. These predictive relationships may prove to be of value in assessment of gait impairments in neurological populations and offer therapeutic targets in intervention trials. The left posterior parietal cortex appeared crucially involved in gait stability during self-paced walking in the real-world and the type of secondary task undertaken may be correlated to frequency-specific coding. These findings validate the employment of measures of trunk acceleration to monitor gait during real-world situations and offer preliminary insights into the neural activity underpinning gait stability.
